# Small DNA Methylation, Big Player in Plant Abiotic Stress Responses and Memory

**DOI:** 10.3389/fpls.2020.595603

**Published:** 2020-12-10

**Authors:** Junzhong Liu, Zuhua He

**Affiliations:** ^1^State Key Laboratory of Conservation and Utilization of Bio-Resources in Yunnan and Center for Life Sciences, School of Life Sciences, Yunnan University, Kunming, China; ^2^National Key Laboratory of Plant Molecular Genetics, CAS Center for Excellence in Molecular Plant Sciences, Shanghai Institute of Plant Physiology and Ecology, Chinese Academy of Sciences, Shanghai, China

**Keywords:** cytosine methylation, *N*^6^-methyladenine DNA methylation, abiotic stress responses, somatic memory, transgenerational inheritance

## Abstract

DNA methylation is a conserved epigenetic mark that plays important roles in maintaining genome stability and regulating gene expression. As sessile organisms, plants have evolved sophisticated regulatory systems to endure or respond to diverse adverse abiotic environmental challenges, i.e., abiotic stresses, such as extreme temperatures (cold and heat), drought and salinity. Plant stress responses are often accompanied by changes in chromatin modifications at diverse responsive loci, such as 5-methylcytosine (5mC) and *N*^6^-methyladenine (6mA) DNA methylation. Some abiotic stress responses are memorized for several hours or days through mitotic cell divisions and quickly reset to baseline levels after normal conditions are restored, which is referred to as somatic memory. In some cases, stress-induced chromatin marks are meiotically heritable and can impart the memory of stress exposure from parent plants to at least the next stress-free offspring generation through the mechanisms of transgenerational epigenetic inheritance, which may offer the descendants the potential to be adaptive for better fitness. In this review, we briefly summarize recent achievements regarding the establishment, maintenance and reset of DNA methylation, and highlight the diverse roles of DNA methylation in plant responses to abiotic stresses. Further, we discuss the potential role of DNA methylation in abiotic stress-induced somatic memory and transgenerational inheritance. Future research directions are proposed to develop stress-tolerant engineered crops to reduce the negative effects of abiotic stresses.

## Glossary

Epigenetics:

The study of relatively stable and inheritable changes in gene expression caused by mechanisms independent of permanent changes in the underlying DNA sequence.

5-methylcytosine (5mC) methylation:

The addition of a methyl group (CH_3_) to the fifth position of the pyrimidine ring of cytosine bases of DNA.

*N*^6^-methyladenine (6mA) DNA methylation:

The addition of a methyl group (CH_3_) to the sixth position of the purine ring of adenine bases of DNA.

RNA-directed DNA methylation (RdDM):

The *de novo* cytosine methylation that involves small interfering RNAs (siRNAs)-generating pathway, long non-coding RNAs (lncRNAs) synthesized by plant-specific RNA Polymerase V (Pol V), chromatin remodeling complex, *de novo* DNA methyltransferase DOMAINS REARRANGED METHYL ASE 2 (DRM2) and a set of DNA or RNA-binding proteins.

Transgenerational epigenetic inheritance:

The transmittance of epigenetic states and associated certain phenotype from one generation to at least the next offspring generation through meiotic cell divisions. The transgenerational epigenetic inheritance may offer the descendants the potential to be adaptive for better fitness.

Somatic memory:

The memories that are mitotically but not meiotically heritable and only last for one generation of organisms.

## Introduction

DNA methylation is a conserved epigenetic modification in eukaryotes and prokaryotes (Law and Jacobsen, 2010; Beaulaurier et al., 2019). In plants, DNA methylation predominantly occurs by the addition of a methyl group to the fifth position of the pyrimidine ring of cytosine bases or the sixth position of the purine ring of adenine bases, which is referred to as 5-methylcytosine [5mC] or *N*^6^-methyladenine [6mA], respectively (Liang et al., 2018a; Zhang H. et al., 2018). The 5mC occurs frequently in all three sequence contexts in plants: the symmetric CG and CHG along with the asymmetric CHH contexts (where H = A, T or C) (Zhang et al., 2006). The DNA methylation levels in plants are different in various species. In *Arabidopsis thaliana*, whole-genome bisulfite sequencing reveals that genome-wide levels of 24% CG, 6.7% CHG and 1.7% CHH contexts are methylated, which predominantly occurs on transposons and other repetitive DNA elements (Cokus et al., 2008). In rice (*Oryza sativa*), the genome-wide DNA methylation level is much higher than *Arabidopsis* with average 44.5% CG, 24.1% CHG, and 4.7% CHH methylation in the two cultivated rice subspecies and their wild ancestors (Li et al., 2012). 5mC of promoter regions usually repress gene transcription, while methylation within the gene body quantitatively impedes transcript elongation in *Arabidopsis* (Zilberman et al., 2007). However, in some genomic regions, two SU(VAR)3-9 homologs, SUVH1, and SUVH3, serve as the methyl reader and recruit two DNAJ domain-containing homologs, DNAJ1 and DNAJ2 to increase the expression of proximal neighboring genes (Harris et al., 2018). 5mC plays important roles in defending the genome against selfish DNA elements and regulating gene expression, which are essential for normal plant growth, development and reproduction as well as appropriate biotic and abiotic stress responses (Zhang H. et al., 2018).

Compared with 5mC, the 6mA abundance in plants is rather lower, ranging from 0.006% to 0.138% in 9-day-old *Arabidopsis* wild-type Col to 0.15–0.55% in rice seedlings (Liang et al., 2018b; Zhang Q. et al., 2018; Zhou et al., 2018). In *Arabidopsis* and rice, 6mA occurs most frequently at plant-specific ANYGA as well as GAGG motifs which is conserved in plantae and animalia (Liang et al., 2018b; Zhang Q. et al., 2018; Zhou et al., 2018). 6mA sites are widely distributed across the *Arabidopsis* genome and 32% of 6mA sites are located within gene bodies, while in rice, 6mA locates at about 20% of genes and 14% of transposable elements (Liang et al., 2018b; Zhou et al., 2018). 6mA seems to be positively associated with gene expression and contributes to plant developments and stress responses (Zhang Q. et al., 2018).

During their immobile lifecycles, plants are exposed to a variety of adverse abiotic stresses, such as drought (water deficiency), salinity (salt), and temperature stresses (heat and cold). These stresses not only inhibit the growth and development of plants, but also pose great threats to crop yield and food safety. Drought and extreme heat have significantly reduced national cereal production by 9–10%, according to the records from the Emergency Events Database and Food and Agriculture Organization of the United Nations during 1964–2007 (Lesk et al., 2016). From 1980 to 2008, global warming has declined the global maize (*Zea mays*) and wheat (*Triticum aestivum*) production by 3.8 and 5.5%, respectively (Lobell et al., 2011). Like other abiotic stresses, cold stress, including chilling stress (0–15°C) and freezing stress (below 0°C), also threatens crop yield and quality, and causes tremendous agricultural yield penalty and economic losses worldwide (Ding et al., 2020). Salinity is another one of the most destructive environmental factors, which affects about 20% of irrigated land and threatens different traits of crop plants, such as the growth rate, photosynthesis, transpiration, yield and quality (Negrão et al., 2017).

To survive in the adverse circumstances, plants employ diverse genetic and epigenetic strategies for regulation of plant growth, development, reproduction and immunity in response to endogenous and exogenous stress signals. The abiotic stress signaling and responses in plant have been extensively studied and recently well summarized (Zhu, 2016; Gong et al., 2020). Plants have evolved quick and sophisticated sensory mechanisms to perceive the abiotic stress cues, convert them to cellular signals and transmit the signals within cells and tissues. So far, several abiotic stress sensors have been identified, such as putative salt sensor glycosyl inositol phosphorylceramide (GIPC) sphingolipids (Jiang et al., 2019), putative cold stress sensor chilling tolerance divergence 1 (COLD1) (Ma Y. et al., 2015), hyperosmotic stress sensor OSCA1 (Yuan et al., 2014), putative heat sensor phytochrome B (phyB) (Jung et al., 2016; Legris et al., 2016), cyclic nucleotide-gated Ca^2+^ channels (CNGCs) (Saidi et al., 2009) and histone variant H2A.Z (Kumar and Wigge, 2010). Upon the perception of abiotic stress signals, these sensors are activated by altering their structure, activity or interacting partners to initiate multilayer downstream stress responses, such as the activation of stress-responsive genes, the regulation of RNA, protein, metabolism and ROS homeostasis. Although the signaling pathways underlying plant responses to different abiotic stresses vary, there are some common theme in the key downstream signaling pathways, such as mitogen-activated protein kinase (MAPK) cascades, G-protein signaling, calcium signaling and hormone signaling (Zhu, 2016).

Besides the significant progress in elucidating the genetic basis of plant abiotic stress responses, great achievements have been made in dissecting the complicated epigenetic regulatory mechanisms in plant adaption to the adverse environments. As one of the most important epigenetic modifications, DNA methylation plays important roles in stress responses in diverse plant species. However, the roles and mechanisms of DNA methylation in plant abiotic stress responses remain largely scattered and fragmented. In this review, we briefly summarize recent progress on the establishment, maintenance and erasing of 5mC and 6mA, and present the divergent roles of DNA methylation in plant responses to different abiotic stresses. Further, we discuss the potential role of DNA methylation in abiotic stress-induced somatic memory and transgenerational inheritance. Finally, we propose some future research directions to breed crops with enhanced stress tolerances.

## DNA Methylation

### 5mC Methylation

#### Establishment of 5mC by the RNA-Directed DNA Methylation Pathway

**FIGURE 1:**
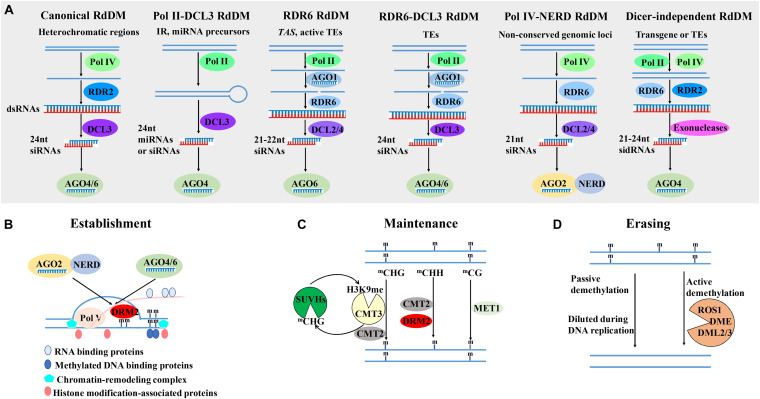
The establishment, maintenance and erasing of 5mC in plants. **(A)** The diverse small RNAs-generating pathways involved in RNA-directed DNA methylation (RdDM). The small RNAs-generating pathways in the canonical Pol IV -RDR2-DCL3-dependent RdDM pathway, Pol II-DCL3 RdDM pathway, RDR6 RdDM pathway, RDR6-DCL3 RdDM pathway, Pol IV-NERD RdDM pathway, and dicer-independent RdDM, are briefly presented, respectively (Cuerda-Gil and Slotkin, 2016). **(B)** The establishment of 5mC in plants. 21–24-nt siRNAs or miRNAs, which are loaded onto AGO2 or AGO4/6, base-pair with Pol V-generated nascent scaffold transcript of target loci, resulting in the DRM2 recruitment and DNA methylation at the source loci with the aid of RNA binding proteins, methylated DNA binding proteins, chromatin-remodeling complex and histone modification-associated proteins (Law and Jacobsen, 2010; Zhang H. et al., 2018). **(C)** The maintenance of 5mC in different contexts. CG, CHG and asymmetric CHH methylation are maintained by MET1, CMT3/CMT2, DRM2/CMT2, respectively. It is needed to note that CMT3 and SUVH4/5/6 form a self-reinforcing feedback loop between ^*m*^CHG and H3K9me (Law and Jacobsen, 2010; Du et al., 2014). **(D)** The erasing of 5mC through active and passive demethylation pathway. In the passive demethylation, 5mC is diluted during DNA replication. In the active demethylation pathway, four bifunctional 5mC DNA glycosylases-apurinic/apyrimidinic lyases, DME, ROS1 and its homologs DML2 and DML3, catalyze the active removal of 5-methylcytosine from all sequence contexts through the base excision repair pathway (Zhang and Zhu, 2012). The proteins are represented in circles. The regulatory pathways are indicated with solid arrows. Abbreviations: mC, methylated cytosine; Pol IV, Polymerase IV; RDR, RNA-DEPENDENT RNA POLYMERASE; DCL, DICER-LIKE; NERD, NEEDED FOR RDR2-INDEPENDENT DNA METHYLATION; AGO, ARGONAUTE; MET1, METHYLTRANSFERASE 1; CMT, CHROMOMETHYLASE; DRM2, DOMAINS REARRANGED METHYLTRANSFERASE 2; SUVH4/5/6, SU(VAR)3-9 HOMOLOGUE; DME, DEMETER; ROS1, REPRESSOR OF SILENCING 1; DML, DME-LIKE 2.

In 1994, for the first time, *de novo* 5mC methylation of genes is found to be induced and targeted by their own RNAs in transgenic tobacco plants infected with viroid (Wassenegger et al., 1994). This phenomenon is described as RNA-directed DNA methylation (RdDM). In the past 26 years, extensive studies have revealed an accumulating knowledge of RdDM. *De novo* 5mC methylation in all sequence contexts is directed by small RNAs and catalyzed by DOMAINS REARRANGED METHYLTRANSFERASE 2 (DRM2) in plants (Zhang H. et al., 2018). The DRM2 activity is regulated by the canonical and non-canonical RdDM pathways, which mainly differs in the small RNAs-generating pathway (Figure 1A; Cuerda-Gil and Slotkin, 2016). Small RNAs are 18–30 nucleotide (nt) non-protein-coding RNAs, which mediate post-transcriptional gene silencing (PTGS) through slicing or translational inhibition, or transcriptional gene silencing (TGS) by targeting chromatin for cytosine or histone methylation. According to their biogenesis and modes of regulation, small RNAs in plants can be divided into two major types: microRNAs (miRNAs) and small interfering RNAs (siRNAs) (Borges and Martienssen, 2015). In the canonical RdDM pathway in *Arabidopsis*, the plant-specific RNA polymerase IV (Pol IV) transcribes heterochromatic regions to generate 30 to 40-nt short RNAs, which are referred to as P4 RNAs (Zhai et al., 2015). RNA-DEPENDENT RNA POLYMERASE 2 (RDR2) then converts P4 RNAs into double-stranded RNAs (dsRNAs) and typically adds an extra untemplated 3′ terminal nucleotide to the second strands. The dsRNAs are processed by RNase III-class endonuclease DICER-LIKE 3 (DCL3) to generate 24- and 23-nt heterochromatic siRNAs (hc-siRNAs) (Singh et al., 2019). The 24-nt hc-siRNAs are exported to the cytoplasm and preferentially incorporated into ARGONAUTE 4 (AGO4) or AGO6, which are re-imported to the nucleus with the help of HEAT SHOCK PROTEIN 90 (HSP90) (Ye et al., 2012). In the nucleus, target loci (mostly transposons and repeats) are transcribed by plant-specific RNA polymerase V (Pol V) to generate non-protein-coding nascent scaffold transcripts, which base-pair with the 24-nt hc-siRNAs by sequence complementarity, resulting in the DRM2 recruitment and DNA methylation at the source loci. A variety of RNA binding proteins, methylated DNA binding proteins, chromatin-remodeling complex and key enzymes responsible for histone H3 lysine 9 dimethylation (H3K9me2) also participate in the establishment of *de novo* DNA methylation (Figure 1B; Zhang H. et al., 2018).

In addition to this canonical Pol IV-RDR2-DCL3-dependent RdDM pathway, several types of non-canonical RdDM pathways have been reported, including Pol II-DCL3 RdDM pathway, RDR6 RdDM pathway, RDR6-DCL3 RdDM pathway, Pol IV- NEEDED FOR RDR2-INDEPENDENT DNA METHYLATION (NERD) RdDM pathway, and dicer-independent RdDM pathway (Figure 1A; Cuerda-Gil and Slotkin, 2016). Pol II transcripts of some inverted repeat (IR) sequences and miRNA precursors can also be cleaved by DCL3 to produce 24-nt small RNAs, which participate in RdDM in *cis* or *trans* (Slotkin et al., 2005; Chellappan et al., 2010; Khraiwesh et al., 2010; Wu et al., 2010). In the RDR6 RdDM pathway, Pol II transcripts of *trans*-acting siRNA (*TAS*) genes and some transcriptionally active transposable elements (TEs) are cleaved by AGO1-bound small RNA-induced silencing complex (RISC), converted into dsRNAs by RDR6, and further cleaved by DCL2/4 into 21–22-nt secondary siRNAs, which are loaded onto AGO6 to initiate RdDM (Wu et al., 2012; Nuthikattu et al., 2013; McCue et al., 2015). High copy number or elevated expression of TEs such as retrotransposon *Evadé* (*EVD*) can also induce the biosynthesis of dsRNAs by RDR6, but such dsRNAs are cleaved by DCL3 to produce 24-nt siRNAs to initiate RdDM, which is referred to as the RDR6-DCL3 RdDM pathway (Marí-Ordóñez et al., 2013). As *EVD* is originally a target of PTGS, the RDR6-DCL3 RdDM pathway may be an important mechanism to silence active TEs when PTGS is saturated (Cuerda-Gil and Slotkin, 2016). In the Pol IV-NERD RdDM pathway, the transcripts of a subset of non-conserved genomic loci are produced by Pol IV but generate 21-nt siRNAs through the sequential roles of RDR6 and DCLs in *Arabidopsis*. The 21-nt siRNAs are loaded to AGO2 and initiate RdDM dependent of NERD, a GW repeat- and PHD finger-containing protein (Pontier et al., 2012). Recently, two groups have reported the dicer-independent RdDM in *Arabidopsis*, in which the dicer-independent siRNAs are generated by distributive 3′–5′ exonucleases (Yang et al., 2015; Ye et al., 2015). In summary, these diverse non-canonical RdDM pathways feed into the canonical RdDM pathways and play subsidiary roles in RdDM pathways.

#### Maintenance of 5mC in Different Contexts

In plants, DNA methylation in three different contexts is maintained by three different pathways. CG, CHG and asymmetric CHH methylation are maintained by METHYLTRANSFERASE 1 (MET1), CHROMOMETHYLASE 3 (CMT3)/CMT2, DRM2/CMT2, respectively (Figure 1C; Zhang H. et al., 2018). In *Arabidopsis*, MET1, ortholog of mammalian DNA methyltransferase DNMT1, is required for the maintenance of CG methylation and normal plant development (Finnegan et al., 1996). During plant mitosis and gametogenesis, MET1 recognizes the hemi-methylated templates and induces the methylation of unmodified CG dinucleotides in the daughter strand (Saze et al., 2003; Zhang H. et al., 2018). The rice genome encodes two closely related putative MET1, OsMET1-1, and OsMET1-2, but only the loss-of-function of OsMET1-2 leads to genome-wide hypomethylation and seedling lethality (Hu et al., 2014). In *Arabidopsis*, VARIANT IN METHYLATION 1-3 (VIM1-3), SRA (SET- and RING-associated) domain methylcytosine-binding proteins, play overlapping roles in the maintenance of global CG methylation in collaboration with MET1 (Woo et al., 2008; Kim et al., 2014).

In the genetic screens for reduced methylation of *Arabidopsis SUPERMAN* locus and *PHOSPHORIBOSYLANTHRANILATE ISOMERASE* (*PAI*), plant-specific methyltransferase CMT3 is found to be indispensable for the maintenance of CHG methylation (Bartee et al., 2001; Lindroth et al., 2001). CMT3-mediated CHG methylation depends on H3K9 histone methyltransferase KRYPTONITE/SUVH4 (KYP) (Jackson et al., 2002). CMT3 and KYP form a self-reinforcing feedback loop between ^*m*^CHG and H3K9me. In the loop, CMT3 is recruited by H3K9me and methylate CHG DNA to create binding sites for KYP and its close homologs SU(VAR)3-9 HOMOLOGUE 5 (SUVH5) and SUVH6; in turn, KYP can methylate H3K9 to generate the binding sites for CMT3 (Figure 1C; Law and Jacobsen, 2010; Du et al., 2014). *Zea* methyltransferase2 (ZMET2) in maize, ortholog of AtCMT3, is also required for ^*m*^CHG (Papa et al., 2001). Crystal structure analysis of ZMET2 and H3K9me2 have revealed that ZMET2 binds H3K9me2 via bromo adjacent homology (BAH) and chromo domains (Du et al., 2012).

CHH methylation is mainly maintained by the DRM2-mediated *de novo* methylation and RdDM pathway (Figure 1C). Besides DRM2, CMT2 mediates CHH methylation at some long TEs through binding to H3K9 methylation (Stroud et al., 2014). Moreover, CMT2 also mediates CHG methylation. Therefore, CMT2, CMT3 and DRM2 collaborate to maintain non-CG methylation, and form self-reinforcing feedback loops with H3K9 methylation (Stroud et al., 2014). DECREASE IN DNA METHYLATION 1 (DDM1), a SWI2/SNF2-like chromatin remodeling enzyme, can facilitate CMT2 to access H1-containing heterochromatin to maintain RdDM-independent CHH methylation (Zemach et al., 2013). In maize, ZmDDM1 regulates the formation of ^*m*^CHH islands through the RdDM pathway (Long et al., 2019). However, in rice, OsDDM1 antagonizes RdDM at heterochromatin and represses non-coding RNA expression from repetitive sequences (Tan et al., 2018), suggesting the distinct roles of DDM1 in different species.

#### Erasing of 5mC Through Active and Passive Demethylation Pathway

In plant growth, development, reproduction and stress responses, 5mC is dynamically regulated by DNA methyltransferases and demethylation pathways. There are two demethylation pathways in plants: passive and active demethylation pathways (Figure 1D). The passive demethylation is a process in which 5mC is diluted from the genome during DNA replication, usually due to the down-regulation of DNA methyltransferase activity or shortage of the methyl donor folate (Zhang H. et al., 2018). In *Arabidopsis* gametogenesis, loss of MET1 in the diploid central cell and the haploid egg cell as well as the loss of DDM1 and Pol IV in the vegetative cell decrease 5mC and strongly reactivate transposons, resulting in the production of siRNAs that may travel to sperm cells or egg cells to reinforce TE silencing (Bourc’his and Voinnet, 2010; Feng et al., 2010).

In the active demethylation pathway, four bifunctional 5mC DNA glycosylases-apurinic/apyrimidinic lyases, DEMETER (DME), REPRESSOR OF SILENCING 1 (ROS1) and its homologs DME-like 2 (DML2) and DML3, have been implicated in the active removal of 5-methylcytosine from all sequence contexts through the base excision repair (BER) pathway (Zhang and Zhu, 2012; Liu and Lang, 2020). ROS1 is the first identified DNA glycosylase/lyase involved in DNA demethylation (Gong et al., 2002). The recruitment of ROS1 to its target genomic regions is mediated by INCREASED DNA METHYLATION (IDM) complex (Zhang H. et al., 2018). Interestingly, the expression of ROS1 is promoted by DNA methylation and a sequence in its promoter functions as a DNA methylation monitoring sequence (MEMS) that senses DNA methylation levels and regulates ROS1 expression to fine-tune genomic DNA methylation (Lei et al., 2015; Williams et al., 2015). DME is preferentially expressed in companion cells of the female and male gametes and initiates active DNA demethylation, which is required for endosperm genomic imprinting and embryo viability (Park et al., 2017).

### *N*^6^-Methyladenine DNA Methylation (6mA)

As a new epigenetic marker in eukaryotes, the establishment, maintenance and erasing of 6mA remain largely obscure. In mammalian, *N*^6^-mA is catalyzed by methyltransferase N6MT1 and removed by 2-oxoglutarate-dependent oxygenase AlkB homolog 1 (ALKBH1) (Xiao et al., 2018; Zhang M. et al., 2020). In rice, OsALKBH1 is proposed to function as 6mA demethylase, as the loss function of OsALKBH1 results in increased 6mA levels (Zhou et al., 2018). 6mA levels are significantly decreased in *Osddm1a*/*ddm1b* double mutants, suggesting that OsDDM1a and OsDDM1b are indispensable for 6mA modification in rice (Zhang Q. et al., 2018). Recent studies have revealed that 6mA DNA modification is positively correlated with gene activation and plays important roles in plant development, and stress responses (Liang et al., 2018b; Zhang Q. et al., 2018; Zhou et al., 2018). For better understanding of the roles of 6mA in plants, it is urgent to identify the key writer and reader of 6mA in plants.

## The Divergent Roles of DNA Methylation in Plant Abiotic Stress Responses

In recent years, multiple technologies have been developed for detecting methylation levels of genome-wide DNA or specific sequence contexts, such as Chop-PCR, methylation sensitive amplification polymorphism (MSAP) technique, methylated DNA immunoprecipitation sequencing (MeDIP-Seq) or 6mA-IP-Seq, and whole genome bisulfite sequencing (WGBS). Using partial digestion by methylation-sensitive restriction enzymes followed by PCR amplification, Chop-PCR can detect the cytosine methylation at the cleavage sites that protects DNA against digestion and therefore can be amplified using PCR (Dasgupta and Chaudhuri, 2019). MSAP is widely applied for analysis of differentially methylated CCGG sites in different plant species with the use of isoschizomers with different methylation sensitivity (such as HpaII and MspI) (Guevara et al., 2017). For MeDIP-Seq and 6mA-IP-Seq, specific antibodies are used to isolate methylated DNA from genomic DNA via immunoprecipitation. WGBS is a sensitive and robust method for genome-wide analysis of 5mC at single-base resolution in plants. These techniques greatly promote the research on the roles of DNA methylation under abiotic stress conditions. The detailed roles of 5mC and 6mA in plant heat, cold, salt and drought stress responses are reviewed as follows.

### Heat Stress

Most plants can only tolerate a certain range of temperature fluctuations. The elevation in temperature, which is 10–15°C beyond the ambient favorable threshold, is referred to as heat stress. There are two-tiered plant tolerance to heat stress: basal and acquired thermotolerance. The basal thermotolerance is an inherent ability for plants to respond and successfully acclimate to heat stress, while acquired thermotolerance means the ability of plants to survive in lethal heat stress after acclimatization to mild heat stress (also known as priming) (Mittler et al., 2012). The thermotolerance in plants are regulated by multiple epigenetic modifications, including DNA methylation (Liu et al., 2015).

**FIGURE 2:**
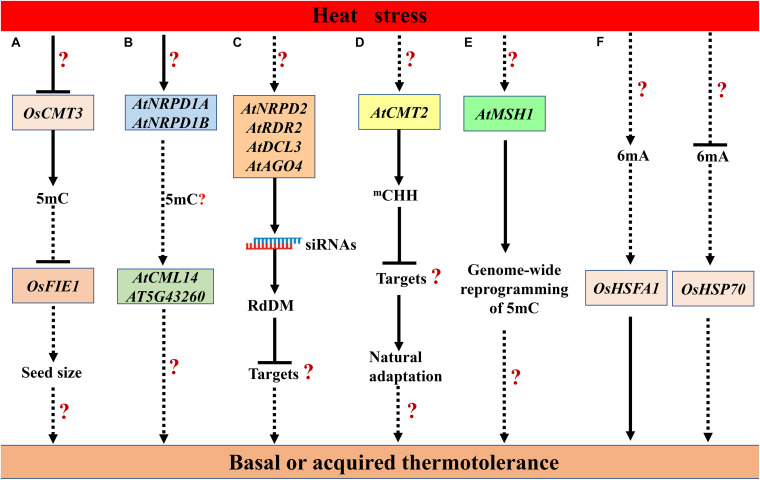
The proposed modulation of heat stress responses by DNA methylation in plants. **(A)** The heat-mediated repression of *OsCHROMOMETHYLASE 3* (*OsCMT3*) partly leads to the up-regulation of *FERTILIZATION-INDEPENDENT ENDOSPERM 1* (*OsFIE1*), which regulates seed size under heat stress in rice (Folsom et al., 2014). **(B)** Heat stress up-regulates *NUCLEAR RNA POLYMERASE D 1A* (*NRPD1A*) and *NRPD1B*, which contributes to heat-induced increase in the expression of *Calmodulin-like 41* (*CML41*) and *At5g43260* (Naydenov et al., 2015). **(C)** The NRPD2, RNA-DEPENDENT RNA POLYMERASE 2 (RDR2), DICER-LIKE 3 (DCL3), and ARGONAUTE 4 (AGO4)-dependent RdDM pathway is required for basal thermotolerance in *Arabidopsis* (Popova et al., 2013). **(D)** The natural variation in *AtCMT2* confers changes in genome-wide CHH-methylation pattern, which contributes to the natural adaptation to variable temperatures (Shen et al., 2014). **(E)** *MutS HOMOLOGUE 1* (*MSH1*)-mediated genome-wide reprogramming may be required for thermotolerance (Virdi et al., 2016). **(F)** Heat stress modulates 6mA levels in rice, and the changes in 6mA levels may contribute to the difference in heat tolerance between Nip and 93-11 through modulating *HEAT SHOCK TRANSCRIPTION FACTOR A 1* (*OsHSFA1*) and *HEAT SHOCK PROTEIN 70* (*OsHSP70*) (Zhang Q. et al., 2018). Genes are shown in boxes. The speculative regulatory paths are shown with broken arrows. Many unknown targets or steps (?) remain to be uncovered in this model.

Heat stress triggers 5mC demethylation globally or at some loci in some plant species. In cotton (*Gossypium hirsutum*) anthers, heat stress (35 to 39°C/29 to 31°C day/night) disrupts the global DNA methylation, especially CHH methylation, in a heat-sensitive line, whereas a heat-tolerant line shows higher methylation level (Min et al., 2014; Ma et al., 2018). The heat-induced down-regulation of *S-ADENOSYL-L-HOMOCYSTEINE HYDROLASE1* (*SAHH1*) and DNA methyltransferases *DRM1*/*3* may contribute to the genome-wide hypomethylation under heat stress (Min et al., 2014). The reduction of DNA methylation may result in the disruption of sugar and reactive oxygen species (ROS) metabolic pathways, leading to microspore sterility (Ma et al., 2018). In soybean (*Glycine max* L.), heat stress (40°C for 3 h) also induces the hypomethylation in all three contexts, especially the ^*m*^CHH, in both root hairs and stripped roots (Hossain et al., 2017). In cultured microspores of *Brassica napus* cv. Topas, heat shock treatment (32°C for 6 h) triggers DNA hypomethylation, particularly in CG and CHG contexts (Li et al., 2016). Another research reveals that after heat stress (37°C for 2 h, and then 45°C for 3 h), more DNA demethylation events occur in the heat-tolerant genotype, while more DNA methylation events occur in the heat-sensitive genotype in *Brassica napus* (Gao et al., 2014). In rice, *OsCMT3* is repressed by heat stress, which may partly lead to the upregulation of *FERTILIZATION-INDEPENDENT ENDOSPERM 1* (*OsFIE1*), a member of Polycomb Repressive Complex 2 (PRC2). The elevated expression of *OsFIE1* may regulate seed size under heat stress (Figure 2A; Folsom et al., 2014). The effect of heat-induced repression of *OsCMT3* on the global 5mC remains to be investigated in rice.

In the model dicot *Arabidopsis thaliana*, the effect of heat stress on key players in 5mC, such as DNA methyltransferases, DNA demethylases, RdDM components, are distinct. These players play diverse roles in thermotolerance through modulating 5mC or other regulatory processes. Heat stress (36°C for 48 h) induced up-regulation of *DRM2*, *NUCLEAR RNA POLYMERASE D 1A* (*NRPD1A*) and *NRPD1B*, the largest subunits of PoI IV and Pol V, respectively. *nrpd1a-1 nrpd1b-1* double mutation abolished DNA methylation in the promoter of *Calmodulin-like 41* (*CML41*) and *At5g43260*, and suppressed their heat-induced increased expression, suggesting the important roles of PoI IV and Pol V in regulating gene expression under heat stress (Figure 2B; Naydenov et al., 2015). *Arabidopsis* plants deficient in *NRPD2*, the common second-largest subunit of PoI IV and Pol V, are hypersensitive to acute heat stress (42°C for 24–34 h). Loss-of-function of RdDM components, RDR2, DCL3 and AGO4 also dramatically decrease the basal thermotolerance (Figure 2C; Popova et al., 2013). In *nrpd2* mutants recovered from heat stress, the misexpression of protein-coding genes, such as auxin-responsive genes, may be affected by their adjacent transposon remnants, which are induced by heat stress (Popova et al., 2013). However, *cmt2* mutant plants and accessions with *CMT2*_*STOP*_ allele display increased tolerance to heat stress (37.5°C for 24 h), natural variation in *CMT2* and associated changes in genome-wide CHH-methylation pattern contribute to the natural adaptation to variable temperatures (Figure 2D; Shen et al., 2014). Some new identified players involved in DNA methylation also play roles in plant heat responses. Depletion of *MutS HOMOLOGUE 1* (*MSH1*) in *Arabidopsis* results in genome-wide reprogramming of DNA methylation (Virdi et al., 2015). Intriguingly, crossing or grafting of the *msh1* mutant to wild type or hemi-complementation of mitochondrial function in the *msh1* mutant can lead to an enhancement of growth vigor and heat tolerance, which may be associated with changes in DNA methylation (Figure 2E; Virdi et al., 2015, 2016). The detailed roles of MSH1 in thermotolerance remain to be investigated. Above all, despite the divergent effects of different key players in 5mC on heat responses, it is no doubt that DNA methylation is important for thermotolerance in *Arabidopsis*.

Heat stress can release the TGS and PTGS of various transgenes and some endogenous loci in *Arabidopsis*, such as exogenous β-*glucuronidase* (*GUS*), 35S promoter of *Cauliflower Mosaic Virus*, endogenous imprinted gene *SDC* and several repetitive elements (transposons and retrotransposons) (Lang-Mladek et al., 2010; Pecinka et al., 2010; Tittel-Elmer et al., 2010; Zhong et al., 2013; Cavrak et al., 2014; Sanchez and Paszkowski, 2014). However, heat-induced activation of these loci occurs without loss of 5mC or seems not to be associated with changes of local 5mC. A *COPIA*-type retrotransposon *ONSEN* can be activated by heat stress and its retrotransposition confers heat-responsiveness to genes close to the new insertion site. In plants deficient in siRNA-biogenesis in RdDM, the heat-induced retrotransposition of *ONSEN* can be transmitted to the unstressed progeny. However, the heat-induced reduction of CHH methylation in *ONSEN* promoter cannot account for the activation of *ONSEN* under heat stress (Ito et al., 2011; Cavrak et al., 2014).

The 6mA levels are positively correlated with heat tolerance in rice (Zhang Q. et al., 2018). Heat stress up-regulates total 6mA levels in both *Japonica* group cultivar Nipponbare (Nip) and *Indica* group cultivar 93-11, and the fold change of 6mA level in 93-11 is 2.6-fold greater than that in Nip. In the signal transduction of heat stress, heat shock transcription factors (HSFs) and heat shock proteins (HSPs) are central players (Mittler et al., 2012). The heat-induced up-regulation of *OsHSFA1* and down-regulation of *OsHSP70* positively correlated with changes in their 6mA levels in 93-11, which may contribute to the more tolerance to heat stress of 93-11 compared with Nip (Figure 2F; Zhang Q. et al., 2018). Whether heat-induced up-regulation of 6mA is conserved in diverse species remains to be elucidated.

In summary, DNA methylation play some noticeable roles in plant heat stress responses. However, the exact roles of DNA methylation in the sensing and signal transduction of heat stress remain unclear in plants. Further studies should pay more attention to the possible roles of DNA methylation in the perception and signaling of heat stress in plants.

### Cold Stress

In plants, the cold signal can be perceived by putative cold sensors, such as the G-protein regulator COLD1 and CBL-INTERACTING PROTEIN KINASE 7 (OsCIPK7) (Ma Y. et al., 2015; Zhang et al., 2019). The PM and ER-localized COLD1 interacts with the RICE G-PROTEIN α SUBUNIT 1 (RGA1) to activate the Ca^2+^ channel and accelerate the influx of extracellular Ca^2+^, which confers chilling tolerance in rice (Ma Y. et al., 2015). OsCIPK7 with a point mutation at the activation loop of the kinase domain exhibits enhanced kinase activity and confers chilling tolerance through Ca^2+^ influx in rice (Zhang et al., 2019). The cold-induced cytosolic Ca^2+^ signal can initiate downstream signaling pathways, such as calcium signaling and MAPK cascade, which regulate the expression of key transcription factors. INDUCER OF CBF EXPRESSION 1 (ICE1), one of the central regulators in plant cold response, activates the C-repeat binding factors/Dehydration-responsive element-binding proteins (CBFs/DREBs), which then binds to the promoter of cold-responsive (COR) genes and actives their expression (Zhu, 2016; Gong et al., 2020). The ICE1-CBF-COR pathway plays a vital role in plant cold stress responses and the pathway is fine-tuned by multiple transcriptional and post-translational processes (Ding et al., 2020).

DNA demethylation has been reported to play important roles in cold stress tolerance in *Arabidopsis*, chestnut (*Castanea sativa Mill.*), poplar (*Populus tremula*), and Cucumber (*Cucumis sativus* L.) (Conde et al., 2017a, b; Lai et al., 2017; Xie et al., 2019). After treated with the DNA methylation inhibitory reagent 5-azacytidine, 30.0–78.3% increases in freezing tolerance are observed in four *Arabidopsis* populations. Similar enhancement of freezing tolerance also occurs in *drm2* mutants (Xie et al., 2019). Cold temperatures induce *CsDML* in chestnut and *PtaDML* in poplar (Conde et al., 2017a, b). In transgenic hybrid poplars overexpressing *CsDML*, apical bud formation is accelerated, alongside with the up-regulation of flavonoid biosynthesis enzymes and accumulation of flavonoids in the SAM and bud scales. The cold stress-mediated up-regulation of *CsDML* may accelerate the bud formation which is required for the survival of the apical meristem under winter (Conde et al., 2017b). In poplar, *PtaDML8/10* knock-down mutants displayed delayed bud break and the targets of *PtaDML*-dependent DNA demethylation are involved in bud break, suggesting the essential roles of chilling-responsive *PtaDML*s in the transition from winter dormancy to shoot growth in woody perennials (Conde et al., 2017a). In Cucumber, cold stress imposes a substantial and global impact on TE-related RdDM, leading to the demethylation of ^*m*^CHH. Besides, cold-induced differentially-methylated regions (DMRs) may be involved in the regulation of genes in ethylene biosynthesis and signaling, which contribute to the temperature-dependent sex determination in cucumber (Lai et al., 2017). However, the loss-of-function of *MSH1* and *RNA-DIRECTED DNA METHYLATION 4* (*RDM4*), an essential player in RdDM pathway, reduce the cold tolerance in *Arabidopsis*. Cold stress poses greater influences on non-CG methylation in *msh1* mutants than in wild-type (Kenchanmane Raju et al., 2018). Surprisingly, RDM4 modulates the cold response by regulating the Pol II occupancy at the promoters of *CBF2/3*, which is independent of RdDM pathway (Chan et al., 2016). Further forward and reverse genetic approaches as well as genome-wide profiling are needed to uncover the roles of DNA methylation-related genes in plant cold stress responses.

**FIGURE 3:**
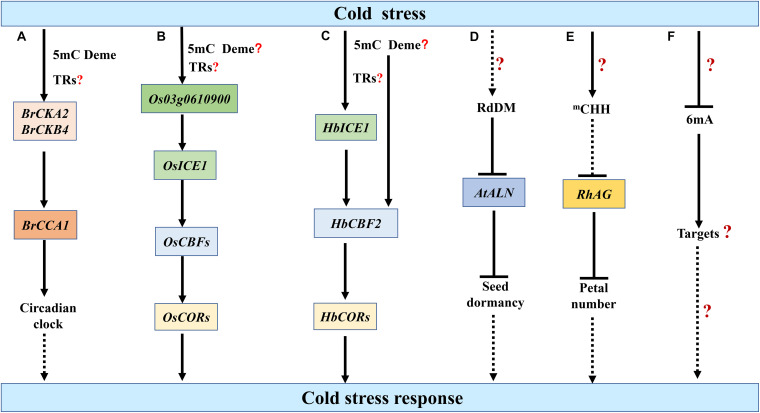
Epigenetic regulation of plant cold stress responses by DNA methylation. **(A)** In *Brassica rapa*, prolonged cold stress mediates the demethylation and increased expression of *CASEIN KINASE II A-SUBUNIT* (*BrCKA2*) and *B-SUBUNIT* (*BrCKB4*), which may regulate cold stress response through the clock gene *CIRCADIAN CLOCK ASSOCIATED 1* (*BrCCA1*) (Duan et al., 2017). **(B)** Cold stress decreases the 5mC in the promoter of *Os03g0610900* and up-regulates its expression, thereby enhancing INDUCER OF CBF EXPRESSION 1 (ICE1)-mediated cold resistance (Guo et al., 2019). **(C)** In *Hevea brasiliensis*, cold stress elevates the transcriptional activities of *HbICE1* as well as *C-repeat binding factor 2* (*HbCBF2*), which may be associated with the DNA demethylation in their promoters (Tang et al., 2018). Whether cold-induced 5mC demethylation results in the up-regulation of *Os03g0610900*, *HbICE1* and *HbCBF2* under cold stress remains to be elucidated **(B,C)**. How cold-induced 5mC demethylation integrated with the activity of transcriptional regulators (TRs) to activate downstream gene expression also needs to be investigated **(A–C)**. **(D,E)** Cold stress up-regulates the 5mC levels of *ALLANTOINASE* (*ALN*) in *Arabidopsis* and *AGAMOUS* homolog *RhAG* in rose (*Rosa hybrida*). The resulting repressed expression of *AtALN* and *RhAG* may be associated with seed dormancy and petal number under cold stress, respectively (Ma N. et al., 2015; Iwasaki et al., 2019). **(F)** Cold stress reduces the 6mA levels in rice, but the roles of 6mA in plant responses to cold stress remain obscure (Zhang Q. et al., 2018). 5mC Deme represents the demethylation of 5mC. The speculative regulatory paths are shown with broken arrows. Many unknown targets or steps (?) remain to be investigated.

Prolonged cold in winter induces the epigenetic silencing of floral repressors, thus ensuring plants overwinter before flowering in spring, a process known as vernalization. Early in 1993, it has been reported that cold-treated *Arabidopsis* plants and *Nicotiana plumbaginifolia* cell line have reduced 5mC in their DNA compared to non-vernalized controls (Burn et al., 1993). However, the cold-induced repression of *FLOWERING LOCUS C* (*FLC*), one of the major determinants of flowering time, is associated with changes of histone methylation but not DNA methylation within the *FLC* locus (Jean Finnegan et al., 2005). In the biennial plant sugar beet (*Beta vulgaris altissima*), the *BvFLC* locus undergoes different regulations of DNA methylation between genotypes that are resistant or sensitive to vernalization-induced bolting, while 5mC at specific cytosines of *VERNALIZATION INSENSITIVE 3* (*BvVIN3*) is correlated with bolting variables (Trap-Gentil et al., 2011). Interestingly, in *Brassica rapa*, vernalization mediates DNA demethylation and increased expression of *CASEIN KINASE II A-SUBUNIT* (*BrCKA2*) and *B-SUBUNIT* (*BrCKB4*), two subunits of the protein kinase CK2. In *BrMET1*-silenced *B.rapa* or plants treated with 5-azacytidine, DNA methylation levels in the promoter of *BrCKA2* and *BrCKB4* are reduced and the expression levels of these two genes increase, suggesting that increased expression of *BrCKA2* and *BrCKB4* could be induced through DNA demethylation. Increased expression of *BrCKA2* and *BrCKB4* confers elevated CK2 activity and results in a shortened period of the clock gene *CIRCADIAN CLOCK ASSOCIATED 1* (*BrCCA1*), which is an important player in perceiving photoperiod (Figure 3A; Duan et al., 2017). However, vernalization-induced demethylation is not a conserved mechanism among species. In hexaploid winter wheat, *VERNALIZATION-A1* (*VRN-A1*) gene, a floral activator in the vernalization pathway, is methylated at CG sites in gene-body region and at non-CG sites in intron 1, which contains fragments of TEs. Vernalization increases the non-CG methylation in intron 1, which can be maintained through mitosis but reset to the pretreated level after sexual reproduction (Khan et al., 2013). Whether such hypermethylation contribute to the vernalization-induced expression of *VRN-A1* remains to be dissected.

The ICE1-CBF-COR pathway is regulated by 5mC DNA methylation, which is associated with cold responses in different species. In crofton weed (*Ageratina adenophora*), the DNA methylation levels in *ICE1* coding region is negatively correlated with the cold tolerance levels among different populations (Xie et al., 2015). *Os03g0610900* is a homologous gene of protein kinase *OPEN STOMATA 1* (*OST1*), which phosphorylates and stabilizes ICE1 under cold stress. Cold stress up-regulates the expression of *Os03g0610900*, thereby enhancing ICE1-mediated cold resistance. The relationship between cold-induced 5mC demethylation in the promoter of *Os03g0610900* and its increased expression needs further investigations (Figure 3B; Guo et al., 2019). In *Hevea brasiliensis*, cold stress elevates the transcriptional activities of *HbICE1* as well as *HbCBF2*, which may be associated with the DNA demethylation in their promoters (Figure 3C; Tang et al., 2018). In *Arabidopsis*, the variation in *ICE1* 5mC methylation likely determines the phenotypic variation in freezing tolerance (Xie et al., 2019). Intriguingly, a recent study reports that a transgene locus harboring a reporter gene in *ice1-1* genome but not the loss-of-function of *ICE1* is responsible for the repression of *DREB1A* expression. The transgene induces hypermethylation in the *DREB1A* promoter through RdDM pathway, which inhibit the transcription of *DREB1A* (Kidokoro et al., 2020). Thus, the ICE1-DREB1A regulatory module in *Arabidopsis* should be validated with other evidences.

Emerging reports have demonstrated that cold stress affects the DNA methylation levels of certain loci in the genome. Under cold stress, the 5mC level in the promoter of *ALLANTOINASE* (*ALN*), a negative regulator of dormancy, is stimulated in a tissue-specific manner through non-canonical RDR6 and AGO6-dependent RdDM pathway, which represses *ALN* expression and further promotes seed dormancy (Figure 3D; Iwasaki et al., 2019). In *Brassica rapa*, cold acclimation decreases the DNA methylation levels in the promoter region of *MITOCHONDRIAL MALATE DEHYDROGENASE* (*BramMDH1*) and up-regulates the expression of *BramMDH1*, which enhances organic acids and photosynthesis to increase heat-tolerance and growth rate in *Arabidopsis* (Liu et al., 2017). In rose (*Rosa hybrida*), cold stress induces CHH methylation of the promoter of *RhAG*, an *AGAMOUS* homolog, which may result in the attenuated expression of *RhAG*. The enhanced suppression of *RhAG* particularly contributes to the cold-mediated increase of petal number (Figure 3E; Ma N. et al., 2015). Interestingly, cold stress can induce stable methylation changes of a non-coding RNA gene and regulate some cold-responsive gene expression in *Populus simonii* (Song et al., 2016).

Unlike heat stress, the 6mA level is significantly decreased in response to cold stress in rice. Following cold stress, the fold change in the 6mA level in Nip is fourfold greater than in 93-11, which may partly explain the higher tolerance of Nip to cold stress than 93-11 (Figure 3F; Zhang Q. et al., 2018). Overall, the roles of 5mC and 6mA in plant responses to cold stress, especially freezing stress, remain largely obscure. DNA methylation play divergent roles in different species under cold stress. High-resolution bisulphite sequencing and in-depth functional analysis are required to improve our understanding on the roles of DNA methylation in plant cold stress responses.

### Salt Stress

**FIGURE 4:**
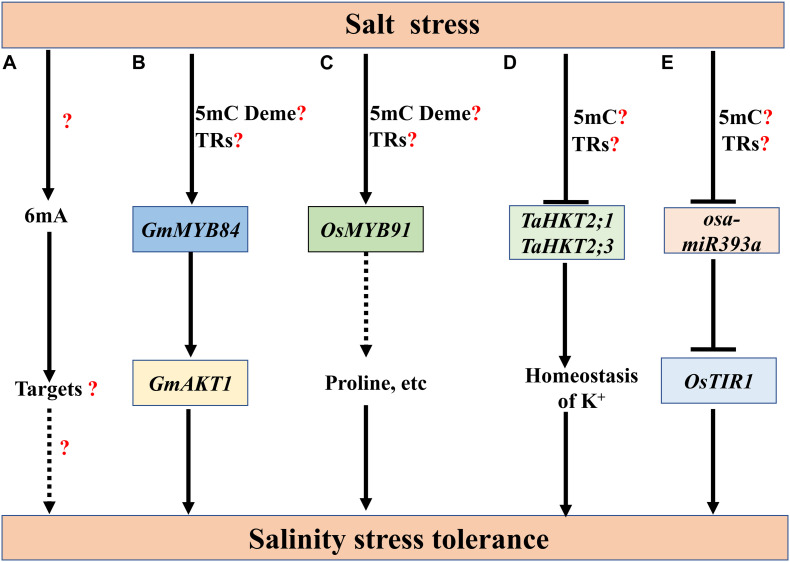
The roles of DNA methylation in plant salt stress responses. **(A)** Salt stress increases the 6mA levels in rice (Zhang Q. et al., 2018), but the regulatory mechanisms underlying 6mA in salt stress remain to be investigated. **(B)** In soybean, salt stress induces 5mC demethylation at the promoter of *MYB DOMAIN PROTEIN 84* (*GmMYB84*), which may be associated with its higher expression. GmMYB84 activates *K^+^ TRANSPORTER 1* (*GmAKT1*) to confer salinity stress tolerance (Zhang W. et al., 2020). **(C)** Salt stress leads to rapid removal of 5mC from the promoter of *OsMYB91*, which may contribute to the salt-induced expression of *OsMYB91*. Plants over-expressing *OsMYB91* show enhanced tolerance with significant increases of proline levels (Zhu et al., 2015). 5mC Deme represents the demethylation of 5mC. **(D)** Salinity stress induces an increase in 5mC levels of *HIGH-AFFINITY POTASSIUM TRANSPORTER 2;1/3* (*TaHKT2;1* and *TaHKT2;3*) that may downregulate their expression, thereby improving the salt tolerance. **(E)** Under salt stress, increased methylation levels of osa-miR393a promoter may lead to a lower expression of osa-miR393a, which may increase the salt tolerance through up-regulation of the target *TRANSPORT INHIBITOR RESPONSE 1* (*OsTIR1*). How salt-induced 5mC methylation or demethylation integrated with the function of transcriptional regulators (TRs) to activate downstream gene expression remains unclear **(B–E)**. The speculative regulatory paths are shown with broken arrows. Many unknown targets or steps (?) remain to be studied.

Similar as heat stress, salt stress also up-regulates 6mA levels in Nip and 93-11. Under salt stress, the 6mA level fold change in 93-11 is 2.5-fold greater than in Nip (Figure 4A) (Zhang Q. et al., 2018). The roles of 6mA in salt stress remain to be studied. Compared with the limited research on 6mA, our knowledge on 5mC in plant salt stress responses has been accumulating. Salt stress induces diverse effects on 5mC in different species. For example, in wheat, salinity stress reduces the 5mC levels in a salinity-tolerant wheat cultivar SR3 and its progenitor parent JN177, which is less tolerant to salt stress. Among the differentially methylated salinity-responsive genes, *TaFLS1*, a flavonol synthase gene, and *TaWRSI5*, a Bowman-Birk-type protease inhibitor, can enhance the salinity tolerance of *Arabidopsis thaliana* (Wang et al., 2014). In soybean root, bisulfite sequencing reveals that 61.2% of CGs, 39.7% of CHG, and 3.2% of CHHs are methylated under durable salt stress, which was slightly lower than those under control condition (Chen et al., 2019). In Rapeseed (*Brassica napus* var. *oleifera*), salinity stress decreases the level of 5mC in the salinity-tolerant cultivar Exagone but increases the methylation levels in the salinity-sensitive cultivar Toccata (Marconi et al., 2013). However, in olive (*Olea europaea*), salt stress induces differentially methylation changes in the 5mC levels of CCGG sites in the tolerant cultivar Royal, which may contribute to plant response to salt stress by slowing down the growth (Mousavi et al., 2019).

The important roles of the key players of 5mC in salt stress tolerance have been limitedly reported. Plants carrying mutation of *RDM16*, which encodes a pre-mRNA-splicing factor 3 and functions in RdDM pathway, are hypersensitive to salt stress in *Arabidopsis* (Huang et al., 2013). *ddm1* and *met1* mutant plants also show high sensitivity to salt stress (Baek et al., 2011; Yao et al., 2012). In *Physcomitrella patens*, *PpDNMT2* accumulates in a temporal manner upon salt stress and *PpDNMT2* knockout plants are unable to recover from salt stress (Arya et al., 2016). Salt stress increases the expression of DNA demethylases in salt-tolerant rice variety Pokkali, which may be linked to the salt-induced demethylation, while in the salt-sensitive variety IR29, the induction of both DNA methyltransferases and demethylases may account for the lower plasticity of DNA methylation. However, the *osdrm2* mutant plants display slight changes of root length and biomass under salt stress as compared to wild-type (Ferreira et al., 2015). Transgenic tobacco overexpressing *AtROS1* displays enhanced tolerance to salt stress, which may be associated with enhanced expression of genes encoding enzymes of the flavonoid biosynthetic and antioxidant pathways (Bharti et al., 2015).

Numerous researches have deciphered the effect of salt stress on the DNA methylation levels of certain loci in the genome. Salt-induced demethylation events at some salt-responsive genes can enhance salt tolerance in different species. In soybean, salt stress markedly reduces the 5mC levels at the promoter of *MYB DOMAIN PROTEIN 84* (*GmMYB84*), which may be associated with its higher expression. GmMYB84 binds to the *cis*-regulatory sequences of *K^+^ TRANSPORTER 1* (*GmAKT1*), thereby conferring salinity stress tolerance (Figure 4B; Zhang W. et al., 2020). Similarly, salt stress leads to rapid removal of 5mC from the promoter of *OsMYB91*, which may contribute to the salt-induced expression of *OsMYB91*. Plants over-expressing *OsMYB91* show enhanced tolerance with significant increases of proline levels and enhanced capacity to scavenge active oxygen (Figure 4C; Zhu et al., 2015). Besides, salinity stress-induced methylation events of some genome loci are also involved in salt tolerance. In the shoot and root of wheat cultivar Kharchia-65, salinity stress induces a genotype- and tissue-specific increase in 5mC levels of *HIGH-AFFINITY POTASSIUM TRANSPORTER 2;1/3* (*TaHKT2;1* and *TaHKT2;3*) that may down-regulate their expression, thereby improving the salt tolerance (Figure 4D; Kumar et al., 2017). Interestingly, at the 2.6 kb upstream of the ATG start codon of *AtHKT1*, a putative small RNA target region is heavily methylated, which inhibits the transcription of *AtHKT1*. The deletion of this region or the loss of 5mC in this region in *met1-3* mutants result in an altered expression pattern of *AtHKT1* and the hypersensitivity to salt stress in plants, suggesting that this putative small RNA target region is essential for maintaining *AtHKT1* expression patterns crucial for salt tolerance (Baek et al., 2011). Under salt stress, the methylation level of osa-miR393a promoter is higher in salt-tolerant genotype FL478 than that of salt-sensitive IR29, which may lead to a lower expression of osa-miR393a in FL478. As salt-responsive osa-miR393a is a negative regulator of salinity stress tolerance in rice, its down-regulation may increase the salt tolerance through up-regulation of the target *TRANSPORT INHIBITOR RESPONSE 1* (*OsTIR1*) (Figure 4E; Ganie et al., 2016). Maize *PROTEIN PHOSPHATASE 2C* (*ZmPP2C*), a negative regulator of ABA signaling, may be repressed by salinity-induced methylation in root, while a positive effector maize *GLUTATHIONE S-TRANSFERASES* (*ZmGST*), may be up-regulated by salinity-induced demethylation in leaf. The salt-induced alteration of 5mC at *ZmPP2C* and *ZmGST* may be involved in maize acclimation to salinity (Tan, 2010). Although salt induces expression changes of some methylated genes or TEs, the roles of salinity-induced methylation or demethylation changes in stress responses remain to be elucidated. For example, in rice, salt, heat and drought stresses can induce the expression of a long terminal repeat (LTR) retrotransposon, *HUO*, which is subjected to RdDM-mediated gene silencing. Multiple *HUO* copies may trigger genomic instability by changing global DNA methylation and small RNA biogenesis, which may result in decreased disease resistance and yield penalty (Peng et al., 2019). *SpPKE1*, a tomato proline-, lysine-, and glutamic-rich type gene isolated from abiotic-resistant species (*Solanum pennellii* LA0716), confers salt tolerance in tomato and tobacco. The detailed roles of heavy methylation in the promoter of *SpPKE1* in plant salt responses remain unclear (Li et al., 2019).

### Drought Stress

The drought-induced up-regulation of 5mC methyltransferases and demethylases has been reported in apple (*Malus* × *domestica* Borkh.), tomato, chickpea (*Cicer arietinum*), barley (*Hordeum vulgare* L.), and eggplant (*Solanum melongena* L.). In chickpea roots, all methyltransferases are up-regulated by drought stress (Garg et al., 2014). Drought stress increases the expression of all cytosine-5-methyltransferases and DNA demethylases except *SmelCMT3a/3b* in leaf tissues of eggplant (Moglia et al., 2019). Similarly, potential DNA methyltransferases and demethylases are induced by drought stress in apple, and *MdCMT2* shows highest induced expression (Xu et al., 2018). *SlDRM6-8*, *SlCMT3* and *SlDNMT2* are significantly induced by dessication in tomato (Kumar et al., 2016). In a drought-tolerant barley cultivar, *HvDME* is also induced by drought stress (Kapazoglou et al., 2013). Further genome-wide analysis of DNA methylation in these species are needed to uncover the roles of drought-induced expressions changes of methyltransferases and demethylases in plant drought responses.

The differential regulation of 5mC methyltransferases and demethylases by drought stress leads to various global methylation changes in diverse species. In *Arabidopsis*, the drought stress-induced hypermethylation partly depends on histone variant H1.3, which can be up-regulated by water deficiency (Rutowicz et al., 2015). Although drought induces changes in DNA methylome in *Arabidopsis*, the methylation changes are unrelated to known transcriptome changes associated with drought stress (Ganguly et al., 2017; Van Dooren et al., 2020). Single-base resolution methylomes analysis in upland cotton by WGBS reveals that drought stress induces hypermethylation in all three sequence contexts, which are almost restored to pre-treatment levels after re-watering (Lu et al., 2017). In *Populus trichocarpa*, drought treatment significantly increases 5mC levels in upstream 2 kb, downstream 2 kb and repetitive sequences (Liang et al., 2014). However, water deficit significantly reduces global 5mC in the model grass *Brachypodium distachyon*, while plants colonized by *Bacillus subtilis* B26 exhibit an overall increase in global DNA methylation under chronic drought, which may attribute to the B26-induced up-regulation of *MET1B-like*, *CMT3-like* and *DRM2-like* genes (Gagné-Bourque et al., 2015). The *Bacillus subtilis* B26-induced methylation changes may be associated with the increased drought stress resilience of *Brachypodium*. Under water-deficiency conditions, the methylation level is high and relatively stable in barley. Drought stress mainly induces new methylations in roots but initiates equal novel methylation and demethylation events in leaves. Such organ-specific methylome changes might regulate the drought resistance in barley (Chwialkowska et al., 2016).

In the model monocot rice, drought stress induces differential 5mC methylation alterations in drought-tolerant variety and drought-sensitive variety (Wang et al., 2011, 2016; Zheng et al., 2013). Under drought conditions, hypermethylation events occur in the drought-susceptible genotypes while drought-tolerant genotypes present hypomethylation behavior (Gayacharan and Joel, 2013). The DMR-associated genes in drought-tolerant introgression line DK151 are mainly involved in stress response, programmed cell death, and nutrient reservoir activity, which may contribute to the constitutive drought tolerance (Wang et al., 2016). Interestingly, a high proportion of multi-generational drought-induced alteration in DNA methylation status is maintained in advanced generations, which may offer the offspring improved drought adaptability in rice (Zheng et al., 2017).

Compared with heat, cold and salt stresses, our understanding regarding the drought stress-induced 5mC changes at drought-responsive TEs and genes is rather limited. Single-base methylome analysis reveals that water deficit is associated with a decrease in CHH methylation in apple cultivars, which may result in the hypomethylated status of TEs (Xu et al., 2018). In tomato, drought stress triggers the activation of a long terminal repeat (LTR) retrotransposon *Rider*, which is controlled by small RNAs and RdDM pathway under normal condition. The drought-induced *Rider* activation might be harnessed to generate genetic and epigenetic variation for crop breeding (Benoit et al., 2019). In a genome-wide association study, a miniature inverted-repeat transposable element (MITE) inserted in the promoter of *ZmNAC111* is identified to be significantly associated with natural variation in maize drought tolerance. Through the RdDM pathway, MITE represses the expression of *ZmNAC111*, which is a positive regulator of drought tolerance in maize (Mao et al., 2015). Drought stress decreases CHH methylation in the regulatory region but increases the CHG and CHH methylation in the coding region of drought-responsive gene *ABSCISIC ACID STRESS RIPENING 2* (*SlAsr2*), which functions in alleviating restricted water availability in tomato roots (González et al., 2013).

## The Role of DNA Methylation in Abiotic Stress Memory

### Somatic Stress Memory

Although abiotic stresses induce various chromatin changes in plants, most epigenetic changes are transient and quickly reset to pre-stressed levels when the abiotic stresses are removed. However, some chromatin changes induced by abiotic stresses can be mitotically heritable and last for several days or even the rest time of plant life in the same generation. In *Arabidopsis*, recurring dehydration stresses result in transcriptional stress memory which is featured by an increase in the rate of transcription and elevated transcript levels of some stress-response genes (Ding et al., 2012). Cold, drought and heat stress treatments can induce somatic abiotic stress memory with a duration of 3–10 days, which mainly involve changes in histone modification, including H3K4me2/me3, H3K27me3 and H3K14ac (Lamke and Bäurle, 2017; Bäurle and Trindade, 2020). The memory of vernalization-induced *FLC* silencing can be maintained in subsequent growth and development under warm temperatures, which is associated with the establishment and maintenance of H3K27me3. In the pro-embryo, the seed-specific transcription factor LEAFY COTYLEDON1 (LEC1) promotes the H3K27me3 demethylation and activation of *FLC*, thereby erasing the vernalization memory (Tao et al., 2017; He and Li, 2018). It seems that DNA methylation is not responsible for the above stress-induced somatic memory. However, in rice, the major portion of salt-induced DNA methylation or demethylation alterations remain after recovery, suggesting that the salinity-induced DNA methylation changes can remember the environmental salt stress and transmit the stress-induced epigenetic states to daughter cells through mitotic cell divisions in the present generation (Wang et al., 2015). It remains a formal possibility that some genome-loci specific 5mC or 6mA changes may function in somatic memory of plant responses to abiotic stresses.

### Transgenerational Inheritance of Stress Memory

Some abiotic stress can induce transgenerational phenotypic changes along with chromatin alterations, which can be detectable until at least one non-stressed generation (Table 1). In *Arabidopsis*, short-wavelength radiation (ultraviolet-C, UV-C) or flagellin treatment increases the frequency of somatic homologous recombination of a transgenic reporter, which persists in the next four untreated generations (Molinier et al., 2006). It is the first report of transgenerational epigenetic inheritance in plants. Since 2006, deciphering the transgenerational memory of plant stress responses has become a fascinating research area. Some stress responses can be only transmitted to the direct progeny, which is termed as intergenerational stress memory, while some stress responses can be memorized for at least two subsequent stress-free generations, which is known as transgenerational stress memory (Lamke and Bäurle, 2017).

**TABLE 1:** Examples of intergenerational and transgenerational stress memory in plants.

Plants	Abiotic stress treatment	Types of stress memory	Major effects	Possible epigenetic regulators	References
*Arabidopsis thaliana*	Ultraviolet-C or flagellin	Transgenerational	Increase in homologous recombination Frequency	Unknown	(Molinier et al., 2006)
*Arabidopsis thaliana* siRNA-biogenesis-deficient plants	37°C for 24 h	Intergenerational	Retrotransposition of *ONSEN*	siRNAs	(Ito et al., 2011)
*Arabidopsis thaliana*	30°C for 14 days	Transgenerational	PTGS release, early flowering and attenuated immunity	H3K27me3 demethylation and siRNAs	(Zhong et al., 2013; Liu et al., 2019)
*Arabidopsis thaliana ddm1mom1* double mutants	37°C for 24 h	Intergenerational	Release of TGS	Altered positioning of nucleosome or others	(Iwasaki and Paszkowski, 2014)
*Arabidopsis thaliana*	50°C for 3 h/day for 5 days	Intergenerational	Fewer but larger leaves, early flowering	DCLs	(Migicovsky et al., 2014)
*Arabidopsis thaliana*	UV-C stress	Transgenerational	Increased transposon expression	DCLs	(Migicovsky and Kovalchuk, 2014)
*Arabidopsis thaliana*	Salt stress for 4 weeks	Intergenerational	Adaption to salt stress	DNA methylation machinery	(Wibowo et al., 2016)
*Arabidopsis thaliana*	42°C for 48 h UV-B stress	Transgenerational	Limited inheritance of TGS release	Histone acetylation	(Lang-Mladek et al., 2010)
*Arabidopsis thaliana*	Successive generations of drought stress	Intergenerational	Increased seed dormancy	Unknown	(Ganguly et al., 2017)
*Arabidopsis thaliana*	Salt, UV-C, cold, heat and flood stress	Intergenerational	Increased homologous recombination frequency	DCLs	(Boyko et al., 2010)
*Arabidopsis thaliana*	Grown at 30°C during reproduction for two generations	Transgenerational	Improved seed production	Unknown	(Whittle et al., 2009)
*Arabidopsis thaliana*	β-aminobutyric acid (BABA) or *PstavrRpt2*	Intergenerational	Improved resistance to biotic stress	Unknown	(Slaughter et al., 2012)
*Arabidopsis thaliana Solanum lycopersicum*	Herbivory, mechanical damage, methyl jasmonate	Transgenerational	Improved resistance to herbivory	siRNAs	(Rasmann et al., 2012)
*Brassica rapa*	42°C for 3 h/day for 7 days	Intergenerational	Fluctuations of smRNAome	miR168 and *braAGO1*	(Bilichak et al., 2015)
*Mimulus guttatus*	Simulated herbivore damage	Intergenerational	Increased trichome density	Unknown	(Scoville et al., 2011)
*Oryza sativa*	Hg^2+^(50 μM/L) for 7 days	Transgenerational	Gene expression changes	DNA methylation	(Ou et al., 2012; Cong et al., 2019)
*Oryza sativa*	Successive generations of drought stress	Transgenerational	Improved drought adaptability	DNA methylation	(Zheng et al., 2017)
*Picea abies*	Daylength and temperature during seed production	Intergenerational	Adaptive plasticity	Unknown	(Johnsen et al., 2005; Kvaalen and Johnsen, 2008)
*Pinus sylvestris* L.	Drought stress	Intergenerational	Tolerant to hot-drought conditions	Unknown	(Bose et al., 2020)
*Polygonum persicaria*	Drought stress	Intergenerational	Improved drought tolerance	Unknown	(Sultan et al., 2009)
*Taraxacum officinale*	Drought and salicylic acid (SA) treatment	Transgenerational	Heritable DNA methylation variation	DNA methylation	(Preite et al., 2018)

The intergenerational stress memory can be triggered by multiple biotic and abiotic stresses, such as flagellin (an elicitor of plant defense), ultraviolet-C, salt, cold, heat and drought stress, β-aminobutyric acid (BABA), methyl jasmonate and the bacteria *Pseudomonas syringae* pv tomato (*PstavrRpt2*) (Table 1; Johnsen et al., 2005; Kvaalen and Johnsen, 2008; Sultan et al., 2009; Boyko et al., 2010; Ito et al., 2011; Scoville et al., 2011; Slaughter et al., 2012; Iwasaki and Paszkowski, 2014; Migicovsky et al., 2014; Bilichak et al., 2015; Wibowo et al., 2016; Ganguly et al., 2017; Bose et al., 2020). Interestingly, in perennial Scots pines (*Pinus sylvestris* L.), environmental memory of naturally dry conditions in the parental trees drive offspring survival and growth under hot-drought conditions (Bose et al., 2020). The stress memory may protect the immediate offspring against recurring stress or offer them the potential for local acclimation to changing environments, while the resetting in the next generation may maximize growth under favorable circumstances (Crisp et al., 2016). The intergenerational stress memory may be mediated by the direct impact of environment factors on the gametogenesis, fertilization and embryo development or maternal cues that are transported into and stored in the seeds when the progeny develops in the mother plants. It remains unclear that how much of the intergenerational stress memory is due to the environment-induced epigenetic changes. The epigenetic regulators involved in the intergenerational stress memory remain largely unidentified, except several reports of the possible roles of small RNAs and DNA methylation (Table 1; Boyko et al., 2010; Ito et al., 2011; Migicovsky et al., 2014; Bilichak et al., 2015; Wibowo et al., 2016). The hyperosmotic stress-induced responses are primarily maintained in the next generation through the female lineage due to widespread DNA glycosylase activity in the male germline, and extensively reset in the absence of stress (Wibowo et al., 2016). How the transient stress memory is maintained during meiosis in the stressed parental plants and removed or reset during the reproduction stage of the next generation remains to be investigated.

Increasing evidences indicate that many abiotic stress responses can exhibit transgenerational epigenetic inheritance (Table 1). Prolonged heat stress can induce transgenerational memory of the release of PTGS and attenuated immunity in *Arabidopsis*, which is mediated by a coordinated epigenetic network involving histone demethylases, heat shock transcription factors and *trans*-acting siRNAs (tasiRNAs) (Zhong et al., 2013; Liu et al., 2019). Cold stress and harsh UV-B treatment-induced release of TGS remain limitedly detectable for two non-stressed progeny generations (Lang-Mladek et al., 2010). The UV-C-mediated activation of some transposons can also be maintained for two generations without the presence of stress, which requires the roles of DCL proteins (Migicovsky and Kovalchuk, 2014). Upon exposure to heavy metal stress, the 5mC state of a Tos17 retrotransposon is altered and shows transgenerational inheritance in rice (Cong et al., 2019). Moreover, heavy metal-transporting P-type ATPase genes (HMAs) are up-regulated under heavy metal stress, which was transgenerationally memorized in the unstressed progeny (Cong et al., 2019). Successive generations of drought stress from the tillering to grain-filling stages induces non-random epimutations and over 44.8% of drought-induced epimutations transmit their altered DNA methylation status to unstressed progeny. Epimutation-related genes directly participate in stress-responsive pathways, which may mediate rice plant’s adaptation to drought stress (Zheng et al., 2017). These transgenerational memories may offer the progeny an adaptive advantage or genomic flexibility for better fitness under diverse abiotic stresses.

Stress-induced transgenerational memory has also been reported in some asexual perennial plants. In the genetically identical apomictic dandelion (*Taraxacum officinale*) plants, various stresses triggered considerable methylation variation throughout the genome, and many modifications were transmitted to unstressed offspring (Verhoeven et al., 2010). In two different apomictic dandelion lineages of the *Taraxacum officinale* group (*Taraxacum alatum* and *T. hemicyclum*) under drought stress or after salicylic acid (SA) treatment, heritable DNA methylation variations are observed across three generations irrespective of the initial stress treatment (Preite et al., 2018). It is needed to note that these stress-induced transgenerational DNA methylation variations in dandelions are genotype and context-specific and not targeted to specific loci (Preite et al., 2018). Unlike most annual plants, the asexual perennial plants use clonal propagation. The stress-induced DNA methylation variations may be largely inherited during mitosis, which may enable the next-generation plants to respond accurately and efficiently to adverse environment factors in some habitats (Latzel et al., 2016). How the methylation variations contribute to the phenotypic variations in asexual perennial plants remains to be investigated.

In the germline and early embryo stage, both the paternal and maternal genomes undergo extensive DNA demethylation via both active and passive demethylation pathways in mammals, which leaves very little possibility for the inheritance of stress-induced changes in methylome (Smith et al., 2012). Some examples of stress-induced transgenerational epigenetic inheritance have been reported in some animals, such as *Caenorhabditis elegans*, the underlying epigenetic marks are mostly histone modifications or small RNAs (Skvortsova et al., 2018). However, the DNA methylation in plants is not erased but rather epigenetically inherited during plant reproduction (Feng et al., 2010; Calarco et al., 2012; Heard and Martienssen, 2014), suggesting a potential role of DNA methylation in transgenerational memory. In the successive generations of *met1-3* mutants deficient in maintaining CG methylation, the loss of mCG is found to progressively trigger new and aberrant genome-wide epigenetic patterns in a stochastic manner, such as RdDM, decreased expression of DNA demethylases and retargeting of H3K9 methylation (Mathieu et al., 2007). Upon potato spindle tuber viroid (PSTVd) infection in tobacco, the body of PSTVd transgene is densely *de novo* methylated in all three contexts. However, in the viroid-free progeny plants, only ^*m*^CG can be stably maintained for at least two generations independent of the RdDM triggers (Dalakouras et al., 2012). Thus, CG methylation may function as a central coordinator to secure stable abiotic transgenerational memory. In a population of epigenetic recombinant inbred lines (epiRILs) with epigenetically mosaic chromosomes consisting of wild-type and *met1-3*, which are nearly isogenic but highly variable at the level of DNA methylation, despite eight generations of inbreeding, unexpectedly high frequencies of non-parental methylation polymorphisms are interspersed in the genome (Reinders et al., 2009). In the F5 individual plants of *ddm1* epiRILs, restoration of wild-type methylation is specific to a subset of heavily methylated repeats targeted by RNA interference (RNAi) machinery (Teixeira et al., 2009). Consistent with this, in the NRPD1 complementation *Arabidopsi*s lines, the DNA methylation of a subset of RdDM target loci can also not be restored even at 20^th^ generations. Many of these non-complemented DMRs overlap with epi-alleles defined in inbreeding experiments or natural accessions, which are functional in plant defense responses (Li et al., 2020). Under salt, drought and increased nutrient conditions in *Arabidopsis thaliana*, *ddm1* epiRILs exhibit phenotypic variations in root allocation, nutrient plasticity, drought and salt stress tolerance (Zhang et al., 2013; Kooke et al., 2015). These reports reinforce the idea that heritable variation in 5mC in epiRILs may allow the generation of epi-allelic variation, which have potential adaptive and evolutionary values. However, while the descendants of drought-stressed *Arabidopsis* lineages exhibit transgenerational memory of increased seed dormancy, the memory is not associated with causative changes in the DNA methylome (Ganguly et al., 2017).

Above all, although the potential roles of epigenetic regulations in transgenerational memory are undoubtable, the roles of stress-induced DNA methylationvariations in the persistence of transgenerational inheritance remain to be further elucidated. The extent to which locus-specific methylation changes might contribute to the maintenance of stress memory also remains unclear. The *de novo* methylation of a particular region can be set up by RdDM and DNA methylation maintenance consolidates RdDM over generations in *Arabidopsis thaliana*, thereby establishing epigenetic memory (Kuhlmann et al., 2014). In *ddm1* epiRILs, several DMRs are identified as bona fide epigenetic quantitative trait loci (QTL^epi^), accounting for 60–90% of the heritability for flowering time and primary root length (Cortijo et al., 2014). Whether the inheritance of DMRs induced by abiotic stress contributes to the transgenerational inheritance requires further investigation. In addition, whether abiotic stresses-induced 6mA changes can be inherited and their roles in stress memory remain elusive.

## Concluding Remarks

Our knowledge on the roles of DNA methylation in plant responses to abiotic stresses is accumulating in recent years. However, these discoveries regarding the roles of 5mC in plant responses to heat, cold, drought and salt stresses are fragmented and scattered (Table 2). The role of 6mA in plant abiotic stress responses is largely unknown. More solid and comprehensive experiments are needed to elucidate the roles the abiotic stresses-induced 5mC and 6mA changes in stress responses through regulating the expression of downstream targets. Besides, it is urgent to investigate how the stress-induced DNA methylation changes recruit or cooperate with other transcriptional regulators to modulate gene expression under abiotic stresses.

**TABLE 2:** The divergent roles of DNA methylation in plant responses to diverse abiotic stresses.

Plants	Abiotic stress	Changes of DNA methylation levels	Major effects	References
*Arabidopsis thaliana*	Heat stress	Altered methylation of transposon remnants	Regulation of basal thermotolerance	(Popova et al., 2013)
*Arabidopsis thaliana*	Heat stress	Changes in genome-wide CHH-methylation pattern	Natural adaptation to variable temperatures	(Shen et al., 2014)
*Arabidopsis thaliana*	Cold stress	Enhanced methylation in *ALN* promoter	Promoting seed dormancy	(Iwasaki et al., 2019)
*Arabidopsis thaliana*	Drought stress	Increased 5mC methylation partly depending on H1.3	Adaptive response to water deficiency	(Rutowicz et al., 2015)
*Arabidopsis thaliana Ageratina adenophora*	Cold stress	Variation in *ICE1* methylation	Cold tolerance divergence in different accessions	(Xie et al., 2015, 2019)
*Brachypodium distachyon*	Drought stress	Decreased global 5mC while *B. subtilis* strain B26 inoculation increases it	Increased drought stress resilience	(Gagné-Bourque et al., 2015)
*Brassica napus*	Heat stress	Increased DNA methylation in heat-sensitive genotype	Adaption to heat stress	(Gao et al., 2014)
*Brassica napus*	Heat stress	DNA hypomethylation	Regulation of heat stress responses in cultured microspores	(Li et al., 2016)
*Brassica napus*	Salt stress	Decreased methylation in the salinity-tolerant cultivar but increased methylation in the salinity-sensitive cultivar	Acclimation to salt stress	(Marconi et al., 2013)
*Brassica rapa*	Cold stress	Decreased DNA methylation levels in the promoter of *BramMDH1*	Increased heat-tolerance and growth rate	(Liu et al., 2017)
*Brassica rapa*	Cold stress	Demethylation of *BrCKA2* and *BrCKB4*	Regulation of floral transition	(Duan et al., 2017)
*Cucumis sativus*	Cold stress	Demethylation at CHH sites	Regulation of temperature-dependent sex determination	(Lai et al., 2017)
*Glycine max*	Heat stress	Hypomethylation in all context	Affecting the expression of genes or TEs under heat	(Hossain et al., 2017)
*Gossypium hirsutum*	Heat stress	Reduced DNA methylation level in heat-sensitive line	Microspore sterility	(Min et al., 2014; Ma et al., 2018)
*Gossypium hirsutum*	Drought stress	Global hypermethylation in all three contexts	Acclimation to drought stress	(Lu et al., 2017)
*Oryza sativa*	Salt, heat and drought stresses	Activation of an LTR retrotransposon, *HUO*	Modulation of stress responses	(Peng et al., 2019)
*Oryza sativa*	Heat, salt, cold stress	Increased 6mA levels in heat and salt stress, decreased 6mA levels in cold stress	Regulation of plant responses to environmental stresses	(Zhang Q. et al., 2018)
*Oryza sativa*	Heat stress	Decreased DNA methylation levels of *OsFIE1*	Regulation of seed size under heat stress	(Folsom et al., 2014)
*Oryza sativa*	Salt stress	Increased methylation level of osa-miR393a promoter	Improved salt tolerance	(Ganie et al., 2016)
*Oryza sativa*	Salt stress	Decreased 5mC levels in the promoter of *OsMYB91*	Enhanced salt tolerance	(Xu et al., 2015)
*Oryza sativa*	Drought stress	Differential 5mC methylation alterations	Constitutive drought tolerance	(Wang et al., 2016)
*Populus trichocarpa*	Drought stress	Increased methylation of upstream and downstream 2 kb, and TEs	Regulation of drought responses	(Liang et al., 2014)
*Rosa hybrida*	Cold stress	Enhanced CHH methylation of the *RhAG* promoter.	Regulation of floral organ development	(Ma N. et al., 2015)
*Solanum lycopersicum*	Salt and drought stresses	Activation of a retrotransposon, *Rider*	Modulation of salt and drought stress responses	(Benoit et al., 2019)
*Solanum melongena*	Salt and drought stresses	Expression changes of C5-MTases and demethylases	Response to salt and drought stresses	(Moglia et al., 2019)
*Triticum aestivum*	Salt stress	Reduced methylation levels in the promoter of salinity-responsive genes	Contribute to the superior salinity tolerance	(Wang et al., 2014)
*Triticum aestivum*	Salt stress	Increased 5mC levels in *TaHKT2;1* and *TaHKT2;3*	Improved salt-tolerance ability	(Kumar et al., 2017)
*Zea mays*	Salt stress	Increased methylation of root *ZmPP2C* and demethylation of leaf *ZmGST*	Acclimation to salt stress	(Tan, 2010)
*Zea mays*	Drought stress	Suppression of *ZmNAC111* by MITE through RdDM	Natural variation in maize drought tolerance	(Mao et al., 2015)

**Box 1. Future research directions.**

• Which enzymes or proteins are responsible for the establishment, maintenance and erasing of 6mA in plants?
• What are the roles of non-canonica RdDM pathways in plant abiotic stress response?
• How are the active and passive demethylation pathways fine-tuned by different abiotic stresses?
• What is the role of 6mA in plant somatic memory and transgenerational memory?
• How to quickly identify QTL^epi^ from epiRILs or epi-mutation library to accelerate investigation on the epigenetic regulation of abiotic stress responses in crops?
• How to efficiently identify the key DMRs responsible for the acclimation to abiotic stresses in plants?
• How are DNA methylation changes integrated with other epigenetic alterations to confer stress tolerance?
• What are the effects of abiotic stresses-induced methylation changes on the expression of key players in the sensing and signal transduction?
• How to manipulate the somatic and transgenerational memory to improve the abiotic stress tolerance of crops without sacrificing growth?

The genome-wide DNA methylation changes induced by abiotic stresses are distinct according to the intensity and duration of stress, the developmental stages, sampled tissues, genotypes and species. The diverse global changes may be attributed to the different impacts of abiotic stresses on the key components of DNA methylation among different species. To improve the tolerance of crops under abiotic stresses, we may pay more attentions to stress-induced DMRs but not the alterations in the global methylome. The mapping of epigenetic quantitative trait loci (QTL^epi^) will greatly accelerate the identification of causal DMRs underlying specific phenotypes or stress tolerance in plants. Several DMRs are identified as QTL^epi^ controlling the variation in growth, morphology and plasticity under normal and saline conditions in *ddm1* epiRILs (Cortijo et al., 2014; Kooke et al., 2015). Linkage-linkage disequilibrium mapping has been used to decipher the QTL^epi^ underlying growth and wood properties in a linkage population and a natural population of *Populus* using MSAP-based analysis (Lu et al., 2020). These QTL^epi^ may be good candidates for engineering plants with better tolerance to abiotic stresses. Interestingly, two recent studies have revealed that *msh1* graft-induced enhanced growth vigor or segregation of an *MSH1* RNAi transgene produced non-genetic memory with multi-generational inheritance (Kundariya et al., 2020; Yang et al., 2020). The *msh1* graft-induced heritable phenotype is RdDM-dependent and requires DCL2-4 to generate siRNAs. In tomato, the *msh1* grafting-enhanced growth vigor in the field can be heritable over five generations, demonstrating the huge agricultural potential of epigenetic variation (Kundariya et al., 2020). The *msh1* memory produced by segregation of an *MSH1* RNAi transgene, also requires RdDM pathway, which involves the function of *HISTONE DEACETYLASE 6* and *MET1*. The *MSH1* RNAi transgene-mediated methylome reprogramming contributes to the phenotypic plasticity in the transgene-free progeny, which may offer them accelerated adaptation to changing environments (Yang et al., 2020).

Our knowledge on the roles of DNA methylation in regulating the signal transduction of abiotic stress is rather limited. Only the ICE1-CBF-COR pathway in the cold signaling has been reported to be regulated by 5mC. The influences of 5mC and 6mA on the HSF-HSP pathway in heat responses and salt-overly-sensitive (SOS) pathway in salt signaling remain to be elucidated. G-protein signaling, MAPK cascades, calcium signaling and hormone signaling are common themes in the key downstream signaling pathways under different abiotic stresses. We still need more efforts to uncover the dynamics of DNA methylation on the important players in these signaling pathways under advert abiotic circumstances.

In recent years, owing to the high efficiency and flexibility, clustered regularly interspaced short palindromic repeats (CRISPR)/CRISPR-associated protein 9 (Cas9) has been widely used in gene editing in various plant species. CRISPR/Cas9 does not induce epigenetic changes on either the target loci, flanking DNA or off-target sites (Lee et al., 2019). A CRISPR/dCas9-based targeted demethylation system comprising the human demethylase TEN-ELEVEN TRANSLOCATION1 (TET1cd) and modified SunTag system has been developed with high specificity and minimal off-target effects, which has been successfully used to target the *FLOWERING WAGENINGEN (FWA)* promoter for demethylation to initiate a heritable late-flowering phenotype (Gallego-Bartolomé et al., 2018). Moreover, TET1-mediated demethylation has been applied for the generation of inheritable 5mC variation through random demethylation of the *Arabidopsis* genome, which results in the expression of previously silenced alleles and uncovers new phenotypic variations (Ji et al., 2018). The fusion of the catalytic domain of 5mC DNA glycosylase ROS1 to dCas9 is also able to reactivate the silenced genes and induce targeted demethylation in a replication-independent manner (Devesa-Guerra et al., 2020). These novel tools open a new window to reactivate expression of previously silenced genes or TEs, and to develop new epi-alleles for improved abiotic stress tolerance. We may take advantage of these tools to introduce epigenetic variations for improving the adaptation to abiotic stress conditions in crops.

As listed in Box 1, many questions concerning the DNA methylation in plant abiotic stress responses remain to be answered by future researches. To further elucidate the role of 6mA in plant abiotic stress responses and memory, one of the most important steps maybe the identification of the 6mA methyltransferases, demethylases and the binding proteins. Plant homologs of mammalian 6mA writers, erasers and readers may be potential targets. Forward genetic screens using reporter lines and reverse genetic approaches such as CRISPR/Cas9 technique will be helpful for identifying proteins involved in the establishment, maintenance and erasing of 6mA in plants. Among the other questions, perhaps the most important question is: how to manipulate the somatic and transgenerational memory to improve the abiotic stress tolerance of crops without sacrificing growth? To address this question, we must identify the key DMRs or QTL^epi^ responsible for the acclimation to abiotic stresses in plants. Systemic screening for DMRs or QTL^epi^ from epiRILs and natural accessions will be powerful approaches (Quadrana and Colot, 2016). The combined application of CRISPR/Cas9 techniques and alternative inducers of DMRs or QTL^epi^ may enable us to engineer crops with enhanced tolerance to abiotic stresses without yield penalty.

## Author Contributions

JL and ZH wrote and revised the manuscript. Both authors have read and approved the final manuscript.

## Conflict of Interest

The authors declare that the research was conducted in the absence of any commercial or financial relationships that could be construed as a potential conflict of interest.
